# Physiotherapy in Your Pocket: Effectiveness of Home Exercises Using an AI-Based Smartphone App for the Postoperative Follow-Up of Hand Injuries – A Randomized, Controlled, Open-Label Study

**DOI:** 10.1055/a-2599-8250

**Published:** 2025-07-02

**Authors:** Simon Bauknecht, Richard Moeller, Martin Mentzel, Michael Lebelt, Jürgen Mack, Daniel Vergote

**Affiliations:** 1 Klinik für Unfall-, Hand-, Plastische und Wiederherstellungschirurgie, Universitätsklinikum Ulm, Ulm, Germany; 2 Praxis, physiotherapie MACK, Ulm, Germany

**Keywords:** Hand therapy, artificial intelligence (AI), fracture, flexor tendon injury

## Abstract

**Introduction:**

Hand injuries can cause considerable functional limitations. Successful surgical treatment requires intensive rehabilitation. However, it is often difficult for patients to obtain timely appointments with a therapist. Several studies have already demonstrated the potential of home exercises. The aim of this study is to investigate the effectiveness of an additional hand therapy app compared to physiotherapy alone.

**Methods:**

This is a prospective, randomized, controlled, open-label study. A total of 112 patients aged 18 to 65 years (MV±SD: 36.9 years±15.5) with metacarpal and finger fractures as well as flexor and extensor tendon injuries participated. The app uses artificial intelligence (AI) and the smartphone’s integrated camera to capture finger movements and determine the range of motion (ROM). Furthermore, the integrated AI automatically adjusts the type and intensity of treatment based on the patient's therapy progress and symptoms. The patients were divided into two groups. The intervention group (IG) received the app Novio Hand for 12 weeks after the immobilization phase in addition to hand therapy (18 units) as standard care (SoC). The control group (CG) received SoC alone. Improvement in ROM was measured at baseline and after 2, 6, and 12 weeks.

**Results:**

Independent t-tests showed significantly greater ROM in the IG compared to the CG at 2 and 6 weeks (p=0.02). A significant trend was observed at 12 weeks. In the IG (50%), significantly more patients achieved the minimal clinically important difference (MCID) of 40 degrees compared to the CG (26%). At 6 weeks, the difference was also significant (IG: 79%, KG: 54%). In the IG, fractures showed an almost full range of motion on average after 6 weeks, whereas in the CG, significant movement deficits could still be quantified after 12 weeks.

**Conclusion:**

Therapy with the Novio Hand app, in addition to SoC can accelerate rehabilitation and improve functional results. The hand therapy app effectively serves as a “physiotherapist” in your pocket, allowing for regular training at any time and from any location.

## Introduction

Hand injuries are among the most common reasons for presentation to emergency departments 1 . Even minor functional impairments can lead to significant limitations in daily life and are often associated with prolonged periods of work incapacity 2 . These injuries are frequently caused by occupational or sports-related accidents 3 4 . The surgical treatment of hand injuries depends on the location and type of injury 4 5 6 . The extent and severity of the injury determine the duration of immobilisation, which may range from a few days to six weeks. However, this necessary immobilisation is also a contributing factor to functional limitations, as longer periods of immobilisation increase the likelihood of adhesion formation, which in turn restricts movement 7 . Therefore, high-quality and sufficiently intensive (hand) therapy is of particular importance in the treatment of hand surgical conditions 2 . In addition to standard care (SoC), which includes physical and occupational therapy, home-based training plays a key role in ensuring the necessary therapeutic intensity in the follow-up phase. The effectiveness of guided home exercise therapy has already been demonstrated in several studies 8 9 . For instance, Gülke et al. (2018) confirmed the efficacy of a structured home-based exercise program for patients undergoing rehabilitation following hand and finger injuries 9 . Despite these promising outcomes, conventional home exercise programs offer little opportunity to monitor therapy adherence – raising the question of how to manage such uncertainty. Digital applications may provide a solution by enabling real-time monitoring while also increasing adherence to therapy.

A systematic literature review revealed a significant research gap in the field of digital hand therapy. Some initial studies exist. For example, Lambert et al. (2017) demonstrated that patients showed greater adherence when using an app-based exercise program compared to a traditional paper-based version 10 . Similar results were reported by Suero Pineda et al. (2016), who observed significant improvements in function, grip strength, and pain levels in favour of the intervention group using a feedback-driven tablet application 11 .

In summary, digital hand therapy has gained considerable relevance in recent years and is now viewed as a promising therapeutic option for patients with hand injuries 12 13 . Compared to conventional rehabilitation, digital hand therapy offers the advantage of immediate availability and independence in terms of time and location. Moreover, therapy content can be tailored to the patient&apos;s individual functional limitations. This study aimed to investigate whether the additional use of an AI-based hand therapy app can lead to more effective rehabilitation in patients with hand injuries compared to SoC alone (i. e., occupational and/or physical therapy).

## Materials and Methods

This study received approval from the responsible ethics committee. It was conducted in accordance with the ethical standards of the Declaration of Helsinki and in compliance with Good Clinical Practice (GCP). All participants provided written informed consent after receiving a comprehensive explanation of the study protocol.

### Study Population

Patients over the age of 18 with a diagnosed metacarpal fracture (S62.3, S62.4), finger fracture (S62.2, S62.6, S62.7), flexor tendon injury (S66.1, S66.6), or extensor tendon injury (S66.3, S66.7) were included in the study. Further inclusion criteria were ownership of a smartphone, willingness to use the app Novio Hand, and sufficient knowledge of the German language. Additionally, due to medico-legal requirements, the app may only be used if the AI model has been sufficiently trained to recognise exceptions to standard patterns, or if the application is specifically approved for certain conditions. Since this was not the case for patients with pronounced skin discoloration (e. g., extensive tattoos or henna designs), use of the app was not permitted in these cases, which led to exclusion. This also applied to the following specific conditions: specific neurological disorders (ataxia, spastic tetraplegia, Parkinson&apos;s disease, epilepsy), untreated uncontrolled infections, skin ulcers affecting the entire fingers/hand/forearm, arthrodeses of finger joints, acute active osteoarthritis of finger joints, advanced osteoporosis, and in certain cases, severely deformed hands or missing limbs.

To ensure a homogeneous and comparable patient cohort, further exclusion criteria were complications such as infections or pseudarthroses, functionally relevant pre-existing conditions or impairments of the hand (e. g., flexion contracture due to Dupuytren&apos;s disease), combination or complex injuries (simultaneous nerve and tendon injuries and fractures), pseudarthroses, outdated fractures (>2 weeks at the time of diagnosis), joint dislocations, and unstable fractures involving the joint. Partial tendon injuries (<50%) without rupture risk were also excluded. Patients who were unable to attend the follow-up assessments T1–T3 within±one week were also excluded.

Recruitment took place over the course of one year (08 Dec 2021–22 Dec 2022). A total of 120 patients were enrolled in the study and randomly assigned equally to both groups. Eight patients did not return for follow-up and were therefore excluded from the study. Ultimately, data from 112 patients (intervention group [IG]=58; control group [CG]=54), aged between 18 and 65 years (mean age 36.92 years±SD 15.46; male: n=82, female: n=30) with metacarpal and finger fractures as well as flexor and extensor tendon injuries were analysed (see Table 1 ).

**Table:** **Table 1** Patient population.

Characteristics		Intervention group	Control group	Group difference
Patients	n	58	54	
Sex	female	17 (29.31%)	13 (24.07%)	p=0.68
	male	41 (70.69%)	41 (75.93%)	
Age (in years)	mean±SD	34.24±14.13	39.41±16.20	p=0.07
**Indications**				
Metacarpal fractures	S62.3, S62.4	13	15	p=0.92
Finger fractures	S62.2, S62.6, S62.7	19	17	
Flexor tendon injuries	S66.1, S66.6	12	11	
Extensor tendon injuries	S66.3, S66.7	14	11	

### Study Design

This was a prospective, randomised, controlled, open-label study with a pre-/post-design. After inclusion in the study and random assignment to the intervention or control group, hand surgical treatment was carried out with indication-specific immobilisation. As soon as the patient was permitted to begin active self-guided exercises, the 12-week intervention period began.

**Fig. 1:**
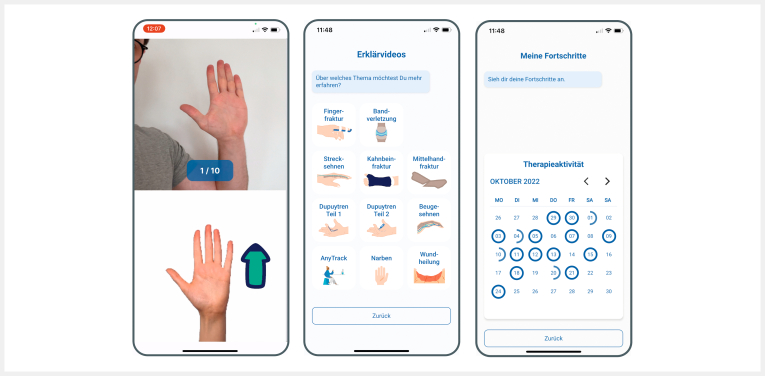
Exemplary images from the Novio Hand app, on the left the AI-supported exercise mode, in the middle the educational elements, on the right the overview of the treatment progress.

In the CG, all patients received SoC consisting of 3×6 units of hand therapy. This followed the criteria defined in the German therapeutic catalogue, with a typical treatment volume of 18 units and a frequency recommendation of 2–3 sessions per week. Patients assigned to the IG additionally received the Novio Hand therapy app during the 12-week intervention period. Novio Hand is an AI-based hand therapy app designed to support patients with hand injuries in independent home training (see Fig. 1 ). It is a certified Class I medical device under MDR with CE marking.

The multimodal hand therapy consists of AI-monitored integrated movement exercises, condition-specific education for hand injuries, and playful elements (gamification), as well as motivational features such as progress tracking, therapy frequency, and push notifications. The movement exercises were developed in collaboration with experienced, certified hand therapy experts (DAHTH hand therapists). During the exercises, the app uses the smartphone camera to detect patients’ movements and qualitatively monitor movement patterns and range of motion in real time. The AI module records changes in mobility and adapts the exercises to the patient’s functional abilities.

In addition, regular prompts by Novio Hand are used before each training session to ensure that the patient’s hand is not being overloaded. For example, patients are asked about their perceived pain intensity before and after exercises using a visual analogue scale (VAS). Pain changes are also queried. Here, the AI works in the background by analysing potential positive or negative changes in pain and adjusting exercise intensity accordingly. During the exercises, the patient receives live visual feedback on the smartphone indicating whether the exercise is being performed correctly. The patient is also motivated to engage in regular training and increase their range of motion through daily reminders, in-app rewards, and the display of personal therapy progress.

In the educational module, patients receive background knowledge on their condition and the healing process through explanatory videos. This aims to enhance the patient’s self-efficacy and therapy adherence. In the gamification module, integrated games are controlled via finger function. A virtual game character (surfer) or rocket must be steered through an obstacle course by repeatedly performing functional exercises (e. g., opening and closing the hand into a fist). At the same time, this promotes improvement in range of motion (ROM), as it becomes increasingly difficult to complete the game successfully with the same ROM. Training frequency can be individually adjusted and tracked via the app, and the patient is reminded of their daily exercises via push notifications.

At both the start of the study (T0) and the follow-up assessments after 2 (T1), 6 (T2), and 12 weeks (T3), finger mobility was quantified by measuring ROM in degrees using the neutral-zero method 9 14 . All measurements were conducted by study physicians using a goniometer following a standardised protocol. The angles of all individual finger joints (metacarpophalangeal, proximal interphalangeal, and distal interphalangeal joints) were recorded and summed to calculate total mobility.

### Data Analysis

Independent t-tests were conducted using Python 3.11.4 for statistical analysis. The general significance level was set at p≤0.05. The effect size (ES) of the t-tests is reported using Cohen’s d, with d<0.5 indicating a small effect, d=0.5–0.8 a moderate effect, and d>0.8 a large effect. Improvement in finger mobility from baseline (∆ to T0) served as the dependent variable, and group assignment (IG, CG) as the independent variable. Analyses were conducted for both the total sample and the individual injury subgroups. In addition, a responder analysis was performed. Differences between the groups with respect to the minimal clinically important difference (MCID) were examined using the non-parametric chi-square test. As no universally accepted MCID value for improvement in hand function was identified in the literature, a change of 15% of the maximum finger ROM (270°) was defined as the MCID for finger function (ROM), based on the value suggested in the methodology paper by the German Institute for Quality and Efficiency in Health Care (IQWiG) 15 . This corresponds to a change of 40°.

## Results

### Range of Motion (ROM)

#### Total population analysis

**Fig. 2:**
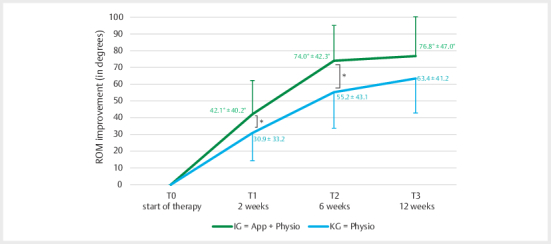
Changes in range of motion (ROM) in the total population. Mean ROM values (mean±SD) for the intervention group (IG) and control group (KG) at T0, T1, T2, and T3. (*p<0.05).

In terms of range of motion (ROM), the IG showed an improvement of 72° by week 6, whereas the CG achieved only 54° over the same period. Independent t-tests of ROM values revealed a significant difference between IG and CG at 2 weeks (T1): t(110)=2.01, p=0.05, Cohen’s d=0.38, and at 6 weeks (T2): t(110)=2.54, p=0.01, Cohen’s d=0.48. More specifically, at T1, the IG demonstrated a markedly greater improvement in ROM (mean±SD: 44.20±37.14; 95% CI: –8.94 to 107.87) compared to the CG (mean±SD: 30.86±33.23; 95% CI: –20.00 to 98.37). This difference persisted at T2, with the IG showing a further ROM increase (mean±SD: 75.47±41.02; 95% CI: –0.75 to 165.00) compared to the CG (mean±SD: 55.22±43.11; 95% CI: 0.81 to 163.50). At 12 weeks (T3), there was a statistical trend toward a group difference in favour of the IG, though this did not reach statistical significance (IG: mean±SD: 78.71±44.92, 95% CI: –6.44 to 167.87; CG: mean±SD: 63.36±41.62, 95% CI: 11.63 to 163.50) (see Fig. 2 ). In summary, the IG showed significantly greater improvements in ROM at both 2 and 6 weeks compared to the CG. At 12 weeks, a statistical trend remained in favour of the intervention.

**Fig. 3:**
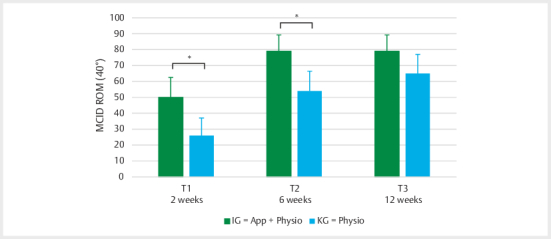
Proportion of patients in the total population who achieved a clinically relevant ROM improvement (MCID=40°) at T1, T2, and T3 (mean±SD). (*p<0.05).

In the responder analysis, 50% (29 patients) in the IG and 26% (14 patients) in the CG achieved an improvement of 40 degrees (MCID) at time point T1 (2 weeks). At T2 (6 weeks), 79% (46 patients) in the IG had already exceeded the MCID, while in the CG only 54% (29 patients) had reached this threshold. At both T1 (2 weeks; p=0.02) and T2 (6 weeks; p=0.01), significantly more patients in the IG reached the MCID compared to the CG. At time point T3 (12 weeks), no significant difference was observed between the two groups (p=0.13) (see Fig. 3 ).

#### Subgroup Analysis

**Fig. 4:**
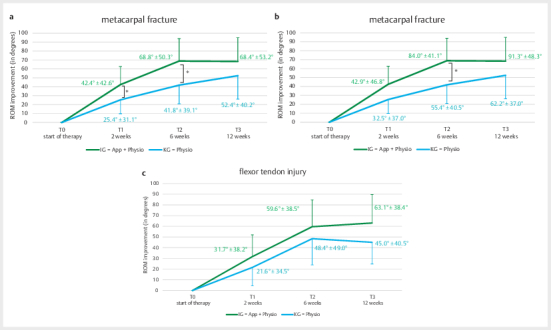
**a** Development of mean ROM values (mean±SD) in patients with metacarpal fractures in IG and CG over the treatment period (T0-T3). **b** Development of ROM changes in patients with finger fractures. Mean values (mean±SD) for IG and CG at the measurement times T0, T1, T2, and T3. **c** ROM development in patients with flexor tendon injuries over time. Representation of the mean values (mean±SD) for IG and CG. (*p<0.05).

The independent t-test analyses for the four injury subgroups revealed a statistically significant difference for metacarpal fractures as early as 2 weeks after the start of the intervention (p=0.02) and again at 6 weeks (T2) (p=0.05). Quantitatively, the IG showed nearly twice the improvement in ROM compared to the CG (T1: IG=mean±SD: 51.83±24.87; CG=25.44±31.12; T2: IG=75.19±45.94; CG=41.78±39.14) (see Fig. 4a ).

For finger fractures, the between-group difference was somewhat smaller, but still statistically significant both at 6 weeks (p=0.04) and 12 weeks (p=0.05). Here, too, the IG outperformed the CG in ROM improvements by roughly one-third on average (T2: IG=83.95±41.07; CG=55.44±40.47; T3: IG=91.32±48.31; CG=62.21±36.97) (see Fig. 4b ).

For flexor and extensor tendon injuries, no statistically significant differences were found between the groups (p>0.05). However, in flexor tendon injuries, the IG still showed greater improvements in ROM – approximately 1.5 times those observed in the CG (see Fig. 4c ).

Extensor tendon injuries generally exhibited the least functional impairments of all patient groups. No relevant differences between groups were detected. Especially by week 6, the IG showed no further improvement compared to the CG, as most patients had already achieved near-complete range of motion, leaving little room for measurable progress.

**Fig. 5:**
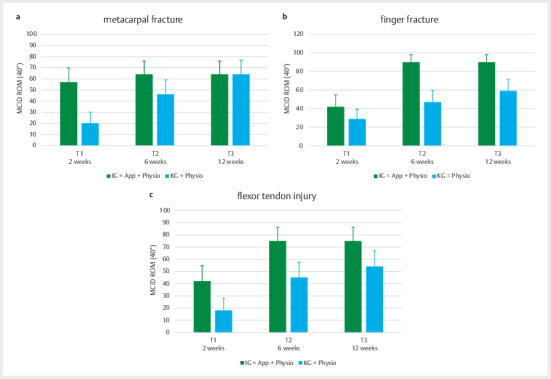
**a** Proportion of patients with metacarpal fractures who achieved an MCID of 40° at T1, T2, and T3. Comparison of IG and CG. **b** Comparison of responder rates in patients with finger fractures with regard to the achievement of MCID (40°) at the measurement time points. **c** Proportion of patients with flexor tendon injuries who achieved a functional ROM improvement (MCID=40°) over the course of treatment. (*p<0.05).

For patients with metacarpal fractures, finger fractures, or flexor tendon injuries, the responder analysis consistently showed that a greater proportion of patients in the IG achieved the MCID of 40 degrees at every follow-up time point compared to the CG. This difference was particularly pronounced early on, with almost twice as many patients in the IG reaching the MCID (see Figs. 5a–c ). A statistically significant group difference based on the chi-square test was observed for finger fractures at 6 weeks (p=0.02) (see Fig. 5b ). A trend in favour of the IG was also observed at 12 weeks (p=0.08), and a similar trend was noted for metacarpal fractures at 2 weeks (p=0.06) (see Fig. 5a ).

## Discussion

It is well established that any injury inevitably leads to scar tissue formation during the healing process. This is particularly relevant in hand surgery, where post-operative scarring often leads to substantial functional impairments. Initially elastic scar tissue contracts and gradually hardens over time, reducing mobility. The extent of movement restriction during scar formation correlates directly with the severity of functional deficits. Therefore, sufficient early mobilisation of the hand is essential to achieve favourable outcomes, irrespective of whether the treatment is surgical or conservative 16 17 18 19 . However, increasing personnel shortages and limited capacity among (hand) therapists often counteract this necessity, leading to prolonged waiting times for patients. In this context, digital home exercise programs represent a cost-effective and efficient alternative. Currently, the digital hand therapy app Novio Hand is reimbursed on a case-by-case basis by the German statutory accident insurance (Berufsgenossenschaft, BG), allowing many patients to benefit from its use. Nationwide coverage by statutory health insurance is planned. The effectiveness of such home-based digital therapy programs has been demonstrated repeatedly in prior research 8 9 , and these findings were confirmed in the present study. Specifically, the IG showed significantly greater improvements in ROM at 2 and 6 weeks after therapy initiation compared to the CG. After 12 weeks, there was still a positive trend favouring the IG. Additionally, significantly more patients in the IG achieved a clinically relevant improvement in finger mobility of at least 40 degrees at weeks 2 and 6 compared to the CG. Subgroup analyses, such as patients with metacarpal fractures, similarly demonstrated significant ROM improvements at 2 and 6 weeks, with a higher proportion of patients achieving a clinically meaningful change in the IG. These results are consistent with those obtained by Then et al. (2020), who observed earlier and faster ROM improvements in the IG receiving gamified therapy during follow-up at weeks 2 and 4 14 . The authors concluded that gamified therapy constitutes a cost-effective and safe alternative to physiotherapy in rehabilitation following metacarpal fractures 14 . Regular performance of functional exercises appears to accelerate the recovery of mobility and significantly shorten the treatment duration. Furthermore, the present results support the findings obtained by Gülke et al. (2018), who investigated the effectiveness of a home exercise program compared to SoC involving physiotherapy. They reported significant effects of home exercise therapy on ROM at 12 weeks post-therapy initiation 9 . This supports the assumption that a digital hand therapy app achieves faster results than a conventional home training program 14 . Since their baseline assessment was conducted at diagnosis, whereas in the present study it was performed at the start of functional exercises, their 12-week time point roughly corresponds to the 6-week assessment here, which further supports the current results 9 . Regarding adherence, Lambert et al. (2017) demonstrated that patient compliance was higher when using a digital app-based exercise program compared to paper-based home exercise instructions 10 . Similarly, Suero Pineda et al. (2023) found improvements in function, grip strength, and pain intensity favouring the IG, who used a feedback-guided tablet program 11 . These findings underline that digital hand therapy as an adjunct in post-injury rehabilitation not only improves functional outcomes but also facilitates earlier return to work and enhances training quality for activities of daily living 4 17 . Patients with finger fractures also showed significant differences favouring the IG at 6 and 12 weeks, with earlier clinically relevant improvements. Finger injuries often involve lymphatic oedema, which can further impair function. Consistent and repeated mobilisation exercises promote lymphatic drainage and reduce swelling, thereby counteracting secondary functional limitations 7 20 . The relatively modest improvements observed in patients with tendon injuries can be explained by the typical post-operative rehabilitation protocols. For flexor tendon injuries, patients are generally restricted to exercises that unload the flexor tendon (e. g., Kleinert protocol) for the first 6 weeks. At this stage, scarring and resulting functional impairments are usually already well established – a known challenge in flexor tendon aftercare. Therefore, it is particularly important to maximise the intensity of permitted therapy. This is corroborated by the findings of Svingen et al. (2021), who reported earlier improvements in the IG using a therapy app compared to SoC in flexor tendon injury patients 21 . In contrast, both groups of patients with extensor tendon injuries improved functionally over 12 weeks without significant intergroup differences, possibly due to the comparatively lower incidence of scar-related adhesions in extensor versus flexor tendon injuries.

In summary, the results demonstrate the potential of the Novio Hand digital therapy app to facilitate earlier and more substantial improvements in ROM in patients with hand injuries. This leads to a marked reduction in rehabilitation time and enables faster reintegration into social and professional life – benefits that are significant both from an individual and socio-economic perspective 2 . Nevertheless, the relatively small sample sizes in the subgroups limit the statistical power of these findings. Further studies with larger cohorts are therefore warranted to validate and extend these results.

## Conclusion

Hand injuries are among the most frequent reasons for presentation in emergency care. Fractures and tendon injuries, in particular, often lead to considerable functional limitations that require early and intensive hand therapy. The findings of this study demonstrate that the use of the hand therapy app Novio Hand leads to significantly greater functional improvements compared to a control group, while simultaneously reducing the overall duration of therapy. The app’s effectiveness can be attributed to its multimodal design, which integrates proven elements such as guided and supervised exercises, gamification, reminder functions, and educational content aimed at enhancing patients’ intrinsic motivation. A central factor for success appears to be the app’s capacity to increase adherence to home exercise regimens – an essential component for functional recovery in terms of mobility and hand function – without diminishing patient motivation. Thus, digital therapy represents a promising adjunct to conventional hand therapy. Wider implementation could contribute to optimising therapeutic outcomes and more efficient utilisation of healthcare resources. Future development of a clinician-facing digital platform to monitor rehabilitation progress and enable timely therapeutic adjustments would be highly beneficial. Given the current limited evidence base, further research is warranted to assess the long-term effectiveness and determine the optimal application of digital therapy modalities in hand injury rehabilitation.
