# Supplementary material for: Physiotherapy in Your Pocket: Effectiveness of Home Exercises Using an AI-Based Smartphone App for the Postoperative Follow-Up of Hand Injuries – A Randomized, Controlled, Open-Label Study
Source: Handchir Mikrochir Plast Chir. 2025 Jul 2;57(3):186–94. doi: 10.1055/a-2599-8250 (PMC12221472; doi:10.1055/a-2599-8250)
Supplement: Related texts

# Research article: Physiotherapie für die Hosentasche: Effektivität von Heimübungen mittels KI-basierter Smartphone App zur Nachbehandlung von Handverletzungen – eine randomisierte, kontrollierte und offene Studie

**Keywords:** Handtherapie, Rehabilitation, Fraktur, Sehnenverletzung, künstliche Intelligenz (KI), Digitale Gesundheitsanwendung (DiGA)

## Abstract

**Fragestellung:**

Verletzungen an der Hand können erhebliche funktionelle Einschränkungen verursachen. Eine erfolgreiche chirurgische Therapie bedarf einer intensiven Nachbehandlung. Für Patienten ist es heutzutage allerdings oft schwierig rechtzeitig Termine beim Therapeuten zu erhalten. In mehreren Studien konnte bereits das Potential von Heimübungen nachgewiesen werden. Im Rahmen der Studie soll die Effektivität einer Handtherapie-App gegenüber alleiniger Handtherapie untersucht werden.

**Methodik:**

In einer prospektiven, randomisierten, kontrollierten Studie wurde die multimodale Handtherapie-App an insgesamt 112 Patienten (MW±SD: 36,9 Jahre±15,5) mit Mittelhand- und Fingerfrakturen sowie Beuge- und Strecksehnenverletzung überprüft. Diese nutzt künstliche Intelligenz, um die Übungen an den Behandlungsfortschritt und die aktuellen Beschwerden der Patienten anzupassen. Die Übungen werden über die integrierte Smartphone-Kamera ausgewertet. Der Bewegungsumfang wird live erfasst, die Anwender erhalten direkt Rückmeldung. Die Studienteilnehmer wurden in zwei Gruppen aufgeteilt. Die Interventionsgruppe (IG) erhielt nach der Ruhigstellungsphase zusätzlich zur Handtherapie (18 Einheiten), als Standardversorgung (SoC), 12 Wochen die KI-basierte Therapie-App Novio Hand. Die Kontrollgruppe (KG) erhielt lediglich SoC. Es wurde die Veränderung der Fingerbeweglichkeit zu Beginn der Therapie sowie nach 2, 6 und 12 Wochen gemessen.

**Ergebnisse:**

Mittels unabhängiger t-tests wurde eine signifikant bessere ROM in der IG im Vergleich zur KG bereits nach 2 und 6 Wochen (p=0,02) festgestellt. Nach 2 Wochen erreichten signifikant mehr Patienten der IG (50%) einen MCID (minimal klinisch bedeutsame Verbesserung) von 40 Grad als Patienten der KG (26%). Nach 6 Wochen war der Unterschied ebenfalls signifikant (IG: 79%, KG: 54%). In der IG konnte bei den Frakturen im Mittel bereits nach 6 Wochen eine nahezu freie Beweglichkeit festgestellt werden, während in der KG auch nach 12 Wochen noch deutliche Bewegungsdefizite quantifiziert werden konnten.

**Schlussfolgerung:**

Die zusätzliche Nutzung einer Handtherapie-App in der Nachbehandlung von Handverletzungen im Vergleich zur Standardversorgung kann die Rehabilitation beschleunigen und das funktionelle Ergebnis verbessern. Die Handtherapie-App ermöglicht Patienten effektiv ein regelmäßiges zeit- und ortsunabhängiges Training. Sie ist quasi ein "Physiotherapeut" für die Hosentasche.

## Einleitung

Handverletzungen gehören zu den häufigsten Gründen für die Vorstellung in der Notfallambulanz 1 . Bereits geringe Funktionseinschränkungen können zu erheblichen funktionellen Defiziten im Alltag führen, welche zudem meist eine lange Arbeitsunfähigkeit nach sich ziehen 2 . Häufig sind solche Verletzungen durch Arbeits- oder Sportunfälle verursacht 3 4 . Die handchirurgische Behandlung richtet sich nach Lokalisation und Art der Verletzung 4 5 6 . Die Art und Schwere der Verletzung bedingt eine gewisse Ruhigstellungszeit, die zwischen wenigen Tagen bis hin zu 6 Wochen schwanken kann. Die notwendige Ruhigstellung ist gleichzeitig auch Teil der Ursache für die Funktionseinschränkungen, da sich mit zunehmender Ruhigstellungszeit vermehrt Adhäsionen ausbilden, welche wiederum zu Funktionseinschränkungen führen 7 . Deshalb ist eine qualitativ gute und quantitativ intensive (hand-) therapeutische Weiterbehandlung bei handchirurgischen Krankheitsbildern von ganz besonderer Bedeutung 2 . Neben der Standardversorgung (SoC) mit Physio- und Ergotherapie, spielt das Heimtraining eine entscheidende Rolle, um die notwendige intensive Nachbehandlung sicherzustellen. Die Effektivität von angeleiteter Heimübungstherapie konnte bereits mehrfach nachgewiesen werden 8 9 . Gülke et al. (2018) konnten die Wirksamkeit eines Heimübungsprogramms bei der Rehabilitation von Patienten mit Hand- und Fingerverletzungen bereits nachweisen 9 . Auch wenn sich die Ergebnisse von Heimübungsprogrammen vielversprechend zeigen, ist eine Überprüfung der Therapietreue bei klassischen Heimübungsprogrammen kaum möglich – so stellt sich die Frage, wie mit solchen Unklarheiten umgegangen wird. Eine Lösung hierfür können digitale Anwendungen sein, mit denen eine Überprüfung möglich gemacht und gleichzeitig die Therapietreue gesteigert wird.

In einer systematischen Literaturrecherche zeigte sich, dass bislang eine erhebliche Forschungslücke auf dem Gebiet der digitalen Handtherapie besteht. Einzelne Arbeiten liegen hierzu bereits vor. So konnten Lambert et al. (2017) zeigen, dass die Adhärenz von Patienten größer war, wenn sie ein Übungsprogramm per App in Vergleich zu einem papierbasierten Heimübungsprogramm durchführten 10 . Ähnliches bestätigten auch Suero Pineda et al. (2016), die Verbesserungen in der Funktion, Griffkraft sowie Schmerzintensität zugunsten der Interventionsgruppe mit einem feedbackgesteuertem Tablet Programm feststellen konnten 11 . Zusammenfassend hat die digitale Handtherapie in den letzten Jahren deutlich an Bedeutung gewonnen und wird als eine vielversprechende Therapieoption für Patienten mit Handverletzungen angesehen 12 13 . Im Vergleich zur konventionellen Rehabilitation bietet die digitale Handtherapie die Möglichkeit, Therapien sofort bereitzustellen und orts- sowie zeitunabhängig durchzuführen. Die Therapie kann individuell gezielt an die Funktionseinschränkungen des Patienten angepasst werden. Im Rahmen dieser Studie sollte überprüft werden, ob durch den zusätzlichen Einsatz einer KI-gesteuerten Handtherapie-App eine effektivere Rehabilitation bei Handverletzungen erreicht werden kann als durch die alleinige Versorgung mittels Handtherapie (Ergo- bzw. Physiotherapie).

## Material und Methoden

Für diese Studie lag ein positives Votum der Ethikkommission vor. Die Studie wurde gemäß den ethischen Standards der Deklaration von Helsinki und nach Good Clinical Practice durchgeführt. Alle Teilnehmer haben nach ausführlicher Aufklärung über die Inhalte der Studie schriftlich eingewilligt.

## Studienpopulation

Patienten über 18 Jahre mit diagnostizierter Mittelhandfraktur (S62.3, S62.4), Fingerfraktur (S62.2, S62.6, S62.7), Beugesehnenverletzung (S66.1, S66.6) und Strecksehnenverletzung (S66.3, S66.7) wurden in die Studie aufgenommen. Weitere Einschlusskriterien waren der Besitz eines Smartphones und die Bereitschaft zur Nutzung der Novio Hand-App sowie ausreichende Kenntnisse der deutschen Sprache. Zusätzlich darf die App aus medicolegalen Gründen nur für jene Indikationen verwendet werden, für welche sie zugelassen ist und wenn die KI ausreichend darauf trainiert wurde, auch Ausnahmen von der Regel zu erkennen. Dies führte zu notwendigen Ausschlüssen von Patienten mit starken Hautverfärbungen (z. B. großflächige Tattoos oder Henna-Bemalungen), stark deformierte Hände oder bei fehlenden Gliedmaßen, ausgedehnte Hautgeschwüre oder unbehandelte/ unkontrollierte Infektionen der Hand, Arthrodesen oder akut aktivierte Arthrosen der Fingergelenke, fortgeschrittene Osteoporose, ebenso spezifische neurologische Erkrankungen (Ataxie, Tetraspastik, M. Parkinson, Epilepsie). Um ein homogenes, vergleichbares Patientenkollektiv zu gewährleisten waren weitere Ausschlusskriterien der Studie allgemeine Komplikationen, wie Infektionen, Pseudarthrosen, Kombinations- u. Komplexverletzungen (gleichzeitiges Vorliegen von Nerven- und Sehnenverletzungen und Frakturen), veraltete Frakturen (>2 Wochen zum Zeitpunkt der Diagnosestellung), Gelenkluxationen sowie instabile Frakturen mit Gelenkbeteiligung und Sehnenteilverletzungen (<50%) ohne Rupturgefahr. Jegliche funktionell relevante Vorschädigungen/ Einschränkungen der Hand (z. B. Beugekontraktur bei M. Dupuytren) waren ebenso ausgeschlossen. Sofern ein Patient nicht+/− innerhalb einer Woche an den Follow-Up Assessments T1–3 teilnehmen konnte, führte dies ebenfalls zum Ausschluss.

Die Rekrutierung erfolgte über ein Jahr (08.12.2021–22.12.2022). Insgesamt wurden 120 Patienten in die Studie eingeschlossen und gleichmäßig auf beide Gruppen randomisiert. 8 Patienten verpassten die Wiedervorstellung, sodass sie von der Studie ausgeschlossen wurden. Letztlich wurden die Daten von112 Patienten (Interventionsgruppe (IG)=58; Kontrollgruppe (KG)=54), im Alter von 18–65 Jahren (MW 36,92 Jahre±SD 15,46, männlich: n=82, weiblich: n=30) mit Mittelhand- und Fingerfrakturen sowie Beuge- und Strecksehnenverletzungen ausgewertet (siehe Tab. 1 ).

**Table:** **Tab. 1** Patientenkollektiv

Charakteristiken		Interventionsgruppe	Kontrollgruppe	Gruppen-unterschied
Teilnehmer	n	58	54	
Geschlecht	weiblich	17 (29.31%)	13 (24.07%)	p=0.68
	männlich	41 (70.69%)	41 (75.93%)	
Alter (in Jahre)	MW±SD	34.24±14.13	39.41±16.20	p=0.07
**Indikationen**				
Mittelhandfrakturen	S62.3, S62.4	13	15	p=0.92
Fingerfrakturen	S62.2, S62.6, S62.7	19	17	
Beugesehnenverletzungen	S66.1, S66.6	12	11	
Strecksehnenverletzungen	S66.3, S66.7	14	11	

## Studienablauf

Es handelt sich um eine prospektive, randomisierte, kontrollierte, offene Studie im prä-/post-Design. Nach Einschluss in die Studie und randomisierter Gruppenzuteilung in IG und KG, erfolgte die handchirurgische Behandlung mit indikationsspezifischer Ruhigstellung. Sobald der Patient mit aktiven eigenständigen Übungen beginnen durfte, begann der Interventionszeitraum von 12 Wochen. In der KG erhielten alle Patienten die SoC mittels 3×6 Einheiten Handtherapie. Diese orientierte sich an den im Heilmittelkatalog definierten Kriterien mit einer orientierenden Behandlungsmenge von 18 Einheiten und einer Frequenzempfehlung von 2–3 Einheiten pro Woche. Patienten, die der IG zugeteilt wurden, erhielten zusätzlich die Handtherapie-App Novio Hand während des Interventionszeitraums von 12 Wochen. Novio Hand ist eine KI basierte Handtherapie-App, um Patienten mit Verletzungen der Hand im eigenständigen Training zu Hause zu unterstützen (siehe Abb. 1 ). Es handelt sich um ein zugelassenes Medizinprodukt der Klasse I nach MDR mit CE-Kennzeichnung. Die multimodale Handtherapie setzt sich aus KI überwachten integrierten Bewegungsübungen, spezifischer Edukation für Handverletzungen und spielerischen Elementen (Gamifizierung) sowie Elementen zur Steigerung der Motivation wie Fortschrittsanalysen, Therapiehäufigkeit und Push-Erinnerungen zusammen. Die Bewegungsübungen wurden zusammen mit erfahrenen ausgebildeten Experten in der Handtherapie (Handtherapeuten der DAHTH) zusammengestellt. Bei den Bewegungsübungen erkennt die App über die Smartphone-Kamera vom Patienten durchgeführte Bewegungsübungen und kann den Bewegungsablauf und -umfang qualitativ in Echtzeit überwachen. Veränderungen im Bewegungsumfang werden hierdurch erfasst und die Bewegungsübungen durch die KI entsprechend den Möglichkeiten des Patienten angepasst. Darüber hinaus werden vor den Übungen regelmäßig Abfragen durch Novio Hand durchgeführt, um zu gewährleisten, dass die Hand des Patienten nicht überlastet wird. So erfolgt beispielsweise vor und nach der Bewegungsübung eine Befragung des Patienten bezüglich der subjektiv empfundenen Schmerzintensität gemäß visueller Analog-Skala (VAS). Zusätzlich werden Veränderungen der Schmerzen abgefragt. Hier wirkt die KI im Hintergrund mit, indem sie mögliche positive wie negative Schmerzveränderungen auswertet und die Übungsintensitäten entsprechend anpasst. Während der Übungen erhält der Patient live visuelle Rückmeldungen auf dem Smartphone, ob die Übung korrekt ausführt wird. Gleichzeitig wird der Patient durch tägliche Erinnerungen, Belohnungen (in der App) und das Anzeigen des persönlichen Therapiefortschritts motiviert die Therapie regelmäßig durchzuführen und den Bewegungsumfang zu steigern. In der Patientenedukation wird dem Patienten durch Erklärvideos Hintergrundwissen zur Erkrankung und zum Heilungsprozess vermittelt. Durch das Wissen über seine Verletzung und den Heilungsprozess soll die Selbstwirksamkeit und Therapieadhärenz des Patienten gesteigert werden. Im Modul der Gamifizierung erfolgt die Steuerung von integrierten Spielen über die Fingerfunktion. Eine virtuelle Spielfigur (Surfer) bzw. Rakete muss durch einen Hindernisparcour gesteuert werden, indem eine Funktionsübung wiederholt wird, beispielsweise durch Öffnen und Schließen der Faust. Gleichzeitig wird hierdurch eine Verbesserung der Range of Motion (ROM) gefördert, da es immer schwieriger wird das Spiel mit demselben Bewegungsumfang erfolgreich zu absolvieren. Die Trainingshäufigkeit kann durch die App individuell angepasst und nachvollzogen werden, über Push-Benachrichtigungen wird der Patient an sein tägliches Training mit der App erinnert.

**Abb. 1:** Beispielhafte Abbildungen aus der App Novio Hand, links der KI gestützte Übungsmodus, mittig die edukativen Elemente, rechts die Übersicht über den Behandlungsfortschritt.

Sowohl zu Beginn der Studie (T _0_ ), als auch zu den Untersuchungszeitpunkten nach 2 (T _1_ ), 6 (T _2_ ) und 12 Wochen (T _3_ ) wurde jeweils die Fingerbeweglichkeit durch Messung der ROM in Grad nach der Neutral-Null-Methode 9 14 quantifiziert. Alle Messungen wurden durch die Studienärzte mittels Goniometer erhoben. Es wurden die einzelnen Gelenkwinkel aller Fingergelenke (Grund-, Mittel- und Endgelenk) quantifiziert und anschließend als Gesamtbeweglichkeit addiert.

## Datenauswertung

Für die statistische Analyse wurden unabhängige t-tests mit Python 3.11.4 durchgeführt. Das allgemeine Signifikanzniveau wurde auf p≤0,05 festgelegt. Die Effektstärke (ES) der t-Tests ist in Cohen´s d angegeben, wobei d<0,5 einen kleinen Effekt, d=0,5–0,8 einen mittelgradigen Effekt und d>0,8 einen großen Effekt darstellt. Die Verbesserung der Fingerbeweglichkeit zur Baseline (∆ zu T _0_ ) diente als abhängige Variable, die Gruppe (IG, KG) als unabhängige Variable. Die Analysen wurden sowohl für die gesamte Gruppe als auch für die einzelnen Verletzungsentitäten berechnet. Darüber hinaus wurde eine Responder-Analyse durchgeführt. Hierzu wurden die Unterschiede zwischen den Gruppen bezüglich der minimal klinisch relevanten Differenz (MCID) mithilfe des nicht parametrischen Chi² Tests untersucht. Da in der Literatur kein allgemeingültiger Wert für die minimal klinisch relevante Verbesserung an der Hand identifiziert werden konnte, wurde in Anlehnung an den im Methodenpapier des IQWIG angegebenen Wert von 15% der Gesamtspannweite 15 eine Veränderung von 15% des maximalen Bewegungsausmaßes der Finger (270°) als MCID für die Fingerfunktion (ROM) definiert. Somit entspricht die MCID einer Veränderung von 40°.

## Ergebnisse

## Range of Motion (ROM)

## Analyse der Gesamtpopulation

Bei der ROM zeigte sich in der IG bis zur 6. Woche eine Verbesserung von 72°, bei der KG von nur 54°. Die Fingerbeweglichkeit wies in der Analyse der ROM-Werte in den unabhängigen t-tests einen signifikanten Unterschied zwischen der IG und KG nach 2 Wochen (T _1_ ) t _(110)_ =2,01, p=0,05, d=0,38 sowie nach 6 Wochen (T _2_ ) t _(110)=_ 2,54, p=0,01, d=0,4819 auf. Im Speziellen wies die IG (MW±SD: 44,20±37,14; KI: − 8,94–107,87) nach 2 Wochen eine merklich markantere Verbesserung der ROM auf als die KG (MW±SD: 30,86±33,23; KI: − 20,00–98,37). Diese Verbesserung zeigte sich ebenfalls nach 6 Wochen (T _2_ ) (IG: MW±SD: 75,47±41,02 KI: − 0,75–165,00; KG: MW±SD: 55,22±43,11 KI: 0,81–163,50). Nach 12 Wochen (T _3_ ) zeigte sich eine statistische Tendenz für einen Gruppenunterschied (IG: MW±SD: 78,71±44,92; KI: − 6,44–167,87; KG: MW±SD: 63,36±41,62; KI: 11,63–163,50) zugunsten der IG (siehe Abb. 2 ). Zusammenfassend zeigte die Interventionsgruppe eine signifikant stärkere Verbesserung der ROM zu den Zeitpunkten 2 und 6 Wochen nach Therapiebeginn im Vergleich zur Kontrollgruppe. Nach 12 Wochen lag nur noch eine nicht-signifikante Tendenz zugunsten der Interventionsgruppe vor.

**Abb. 2:** Veränderungen des Bewegungsausmaßes (ROM) in der Gesamtpopulation. Mittlere ROM-Werte (MW±SD) für die Interventionsgruppe (IG) und Kontrollgruppe (KG) zu T0, T1, T2 und T3. (*p<0,05).

Bei der Responderanalyse wiesen 50% (29 Patienten) der IG und 26% (14 Patienten) der KG zum Zeitpunkt T _1_ (2 Wochen) eine Veränderung von 40 Grad (MCID) auf. Zum Zeitpunkt T _2_ (6 Wochen) waren es mit 46 Patienten in der IG bereits mehr als dreiviertel (79%), wogegen mit 29 Patienten (54%) in der KG erst knapp die Hälfte über dem MCID von 40° lagen. Sowohl bei T _1_ nach 2 Wochen (p=0,02) als auch nach 6 Wochen (T _2_ ) (p=0.01) erreichten signifikant mehr Patienten der IG die MCID als Patienten der KG. Zum Zeitpunkt T _3_ liegt kein signifikanter Unterschied zwischen den beiden Gruppen vor (p=0,13) (siehe Abb. 3 ).

**Abb. 3:** Anteil der Patienten in der Gesamtpopulation, die eine klinisch relevante ROM-Verbesserung (MCID=40°) zu T1, T2 und T3 erreicht haben (MW±SD). (*p<0,05)

## Subgruppenanalyse

Die Analysen der unabhängigen t-tests für die vier Verletzungsentitäten ergaben für die Mittelhandfraktur bereits einen signifikanten Unterschied 2 Wochen nach Interventionsbeginn (p=0,02) sowie zu T _2_ (p=0,05) nach 6 Wochen. Quantitativ zeigte die IG (T _1_ =MW±SD: 51,83±24,87; T _2_ =MW±SD: 75,19±45,94) durchschnittlich fast doppelt so starke Verbesserungen in der ROM als die KG (T _1_ =MW±SD: 25,44±31,12; T _2_ =MW±SD: 41,78±39,14) (siehe Abb. 4a ).

**Abb. 4:** **a** Entwicklung der mittleren ROM-Werte (MW±SD) bei Patienten mit Mittelhandfrakturen in IG und KG über den Behandlungszeitraum (T0–T3). **b** Verlauf der ROM-Veränderungen bei Patienten mit Fingerfrakturen. Mittlere Werte (MW±SD) für IG und KG zu den Messzeitpunkten T0, T1, T2 und T3. **c** ROM-Entwicklung bei Patienten mit Beugesehnenverletzungen im Zeitverlauf. Darstellung der mittleren Werte (MW±SD) für IG und KG. (*p<0,05)

Bei den Fingerfrakturen fiel der Unterschied etwas geringer aus, dennoch konnte auch hier ein signifikanter Unterschied sowohl nach 6 (p=0,04) als auch nach 12 Wochen (p=0,05) festgestellt werden (IG: T _2_ =MW±SD: 83,95±41,07; T _3_ =91,32±48,31; KG: T _2_ =MW±SD: 55,44±40,47; T _3_ =62,21±36,97). Die Verbesserung der ROM fiel in der IG um durchschnittlich ein Drittel besser aus als in der KG (siehe Abb. 4b ).

Bei den Beuge- und Strecksehnenverletzung lagen keine statistisch signifikanten Unterschiede zwischen den Gruppen vor (p>0,05). Allerdings fiel auf, dass auch bei Beugesehnenverletzungen die IG eine stärkere Verbesserung aufwies als die KG. So verbesserte sich die ROM der Patienten in der IG um das 1,5-fache im Vergleich zur KG (siehe Abb. 4c ).

Strecksehnenverletzungen wiesen prinzipiell die geringsten Funktionseinschränkungen aller Patienten auf. Hier konnten keine relevanten Unterschiede zwischen den Gruppen festgestellt werden. Die IG zeigte vor allem nach 6 Wochen keine relevanten Verbesserungen mehr im Vergleich zur KG, da sie bereits größtenteils eine freie Beweglichkeit aufwiesen und somit keine weiteren Verbesserungen mehr möglich waren.

Bei den Verletzungsentitäten Mittelhandfrakturen, Fingerfrakturen oder Beugesehnenverletzungen zeigte die Responderanalyse, dass zu jedem Zeitpunkt mehr Patienten der IG die MCID von 40 Grad erreichten als Patienten der KG. Vor allem zu Beginn fiel der Unterschied am stärksten aus mit nahezu doppelt so vielen Patienten (siehe Abb. 5c ). Signifikante Unterschiede nach dem Chi²-Test liegen bei Fingerfrakturen nach 6 Wochen (p=0,02) vor (siehe Abb. 5b ). Nach 12 Wochen zeigte sich ein tendenzieller Vorteil in der IG (p=0,08). Ebenso zeigte sich bei Mittelhandfrakturen ein tendenzieller Vorteil in der IG nach 2 Wochen (p=0,06) (siehe Abb. 5a ).

**Abb. 5:** **a** Anteil der Patienten mit Mittelhandfrakturen, die eine MCID von 40° zu T1, T2 und T3 erreicht haben. Vergleich von IG und KG. **b** Vergleich der Responder-Raten bei Patienten mit Fingerfrakturen hinsichtlich des Erreichens der MCID (40°) zu den Messzeitpunkten. **c** Anteil der Patienten mit Beugesehnenverletzungen, die eine funktionelle ROM-Verbesserung (MCID=40°) im Verlauf der Therapie erzielt haben. (*p<0,05)

## Diskussion

Bekanntlich führt jede Verletzung im Rahmen des Heilungsprozesses zur Bildung von Narbengewebe. Gerade bei handchirurgischen Erkrankungsbildern – insbesondere nach operativen Eingriffen – führen diese Narben häufig zu erheblichen Funktionseinschränkungen an der Hand, denn das initial noch elastische Narbengewebe kontrahiert und verfestigt sich im Verlauf zunehmend. Je geringer die Bewegung und der Bewegungsumfang im Rahmen des Vernarbungsprozesses ausfällt, desto stärker kann das Funktionsdefizit ausfallen. Um ein gutes Outcome zu erzielen ist eine suffiziente frühzeitige Mobilisation der Hand essentiell, unabhängig davon, ob ein operativ oder konservatives Vorgehen gewählt wurde 16 17 18 19 . Dies wird jedoch durch den zunehmenden Personalmangel und fehlende Kapazitäten bei (Hand-)Therapeuten konterkariert, was oft zu langen Wartezeiten bei Patienten führt. Eine kostengünstige und gleichzeitig effektive Alternative stellen digitale Heimübungsprogramme dar. Aktuell wird die digitale Handtherapie mit der untersuchten Handtherapie-App Novio Hand per Einzelfallentscheidung von der Berufs-Genossenschaft (BG) übernommen, sodass zahlreiche Patienten bereits erfolgreich therapiert werden konnten. Eine flächendeckende Erstattung durch die gesetzlichen Krankenkassen ist geplant. Die Effektivität solcher Heimübungsprogramme konnte jedoch bereits mehrfach nachgewiesen werden 8 9 . Diese Ergebnisse konnten im Rahmen dieser Studie bestätigt werden. So zeigte die IG eine signifikant stärkere Verbesserung der ROM 2 und 6 Wochen nach Therapiebeginn im Vergleich zur KG. Nach 12 Wochen lag noch eine Tendenz zugunsten der IG vor. Darüber hinaus zeigten signifikant mehr Patienten der IG eine klinisch relevante Verbesserung der Fingerbeweglichkeit von 40 Grad als Patienten der KGe nach 2 und nach 6 Wochen. Auch bei der Analyse der Subgruppen zeigten beispielsweise Patienten der IG mit Mittelhandfrakturen signifikante Verbesserungen der ROM nach 2 und 6 Wochen. Zudem wiesen mehr Patienten dieser IG eine klinisch relevante Veränderung auf. Diese Ergebnisse gehen einher mit Ergebnissen von Then et al. (2020), bei denen die Patienten in der IG eine frühere und schnellere Verbesserung der ROM während der Nachbeobachtung in der zweiten und vierten Woche zeigten 14 . Laut Autoren ist die in der IG angewendete gamifizierte Therapie eine kostengünstige und sichere Alternative zur Physiotherapie in der Rehabilitation nach einer Mittelhandfraktur 14 . Vor allem die regelmäßige Anwendung von Funktionsübungen scheint zu einer frühzeitigen Verbesserung der Beweglichkeit zu führen und somit den Behandlungszeitraum wesentlich zu verkürzen. Weiterhin unterstützen die vorliegenden Befunde die Ergebnisse von Gülke et al. (2018), die die grundsätzliche Wirksamkeit eines Heimübungsprogramms im Vergleich zur SoC mit Physiotherapie untersucht haben. Die Ergebnisse zeigten 12 Wochen nach Therapiebeginn signifikante Effekte der Heimübungstherapie auf die ROM 9 . Dies unterstützt die Annahme, dass eine digitale Handtherapie-App schnellere Ergebnisse erzielt als ein herkömmliches Heimübungsprogramm 14 . Bei Gülke et al. (2018) war die Baseline-Erhebung bei Diagnosestellung und nicht, wie in dieser Studie, zu Beginn der funktionellen Beübung. Somit entspricht der 12 Wochen Zeitpunkt durchschnittlich dem 6 Wochen Zeitpunkt in dieser Studie, was die Ergebnisse dieser Studie unterstützt 9 . In Bezug zur Heimübungstherapie zeigten Lambert et al. (2017) in einer Studie, dass die Adhärenz von Patienten größer war, wenn sie ein Übungsprogramm mittels digitaler App in Vergleich zu einem papierbasierten Heimübungsprogramm durchführten 10 . Ähnliches bestätigten auch Suero Pineda et al. (2023), die Verbesserungen in der Funktion, Griffkraft sowie Schmerzintensität zugunsten der IG mit einem feedbackgesteuertem Tablet Programm feststellen konnten 11 . Die zusätzliche Anwendung digitaler Handtherapie in der Nachbehandlung von Handverletzungen führt somit zu einem verbesserten funktionellen Outcome. Dies ermöglicht auch eine schnellere Wiedereigliederung ins Berufsleben und verbessert die Qualität des Trainings für Aktivitäten des täglichen Alltags 4 17 .

Auch die Subgruppe der Fingerfrakturen wies signifikante Unterschiede nach 6 und 12 Wochen zugunsten der IG bei erneut signifikant früherer klinisch relevanter Veränderung auf. Gerade bei Fingerverletzungen führen die häufig vorliegenden Lymphödeme zu weiteren Funktionseinschränkungen. Durch wiederholtes und konsequentes Beüben der betroffenen Hand kann der Lymphabfluss sowie die damit verbundenen Abschwellungen der Hand gezielt gefördert 7 20 und damit drohenden Funktionseinschränkungen zusätzlich entgegen gewirkt werden.

Die geringer ausfallenden Verbesserungen bei Sehnenverletzungen lassen sich dadurch erklären, dass der Patient in den ersten 6 Wochen nach der operativen Versorgung durch die klassische Nachbehandlung nach Kleinert nur Übungen unter Entlastung der Beugesehne durch Zügelung durchführen konnte. Der Vernarbungsprozess und die damit einhergehenden Funktionseinschränkungen zeigen sich zu diesem Zeitpunkt schon deutlich manifestiert – ein altbekanntes Problem insbesondere in der Nachbehandlung von Beugesehnenverletzungen. Umso wichtiger erscheint uns, dass das, was an Therapie durchgeführt werden darf, auch so intensiv wie möglich erfolgt. Die Untersuchung von Patienten mit einer Beugesehnenverletzung konnte ebenfalls eine früher einsetzende Verbesserung in der IG im Vergleich zur KG bestätigen, was sich mit den Ergebnissen einer randomisiert-kontrollierten Studie von Svingen et al. (2021) deckt 21 .

Bei Patienten mit einer Strecksehnenverletzung verbesserten sich beide Gruppen in ihrer Funktion innerhalb des Untersuchungszeitraums von 12 Wochen. Ein signifikanter Unterschied zwischen den Gruppen konnte nicht festgestellt werden. Möglicherweise lässt sich dies mit dem verhältnismäßig geringeren Auftreten von narbigen Adhäsionen im Vergleich zu Beugesehnenverletzungen erklären.

Zusammenfassend zeigen die Ergebnisse das Potenzial der digitalen Handtherapie-App Novio Hand. Die App trägt zu einer frühzeitigen und größeren Verbesserung der ROM bei Patienten mit Handverletzungen bei, wodurch die Rehabilitationszeit deutlich verkürzt und ein früherer Einstieg in das soziale und berufliche Leben ermöglicht wird. Diese verkürzte Rehabilitationszeit kann aus individueller sowie volkswirtschaftlicher Sicht von hoher Bedeutung sein 2 . Trotz der vielversprechenden Ergebnisse sollte beachtet werden, dass die einzelnen Subgruppen relativ klein waren. Dies kann die statistische Aussagekraft der Ergebnisse einschränken. Aufgrund dessen sollten weitere Studien mit größerer Fallzahl durchgeführt werden, um die Ergebnisse zu validieren.

## Schlussfolgerung

Die Ergebnisse dieser Studie zeigen, dass die Anwendung der Handtherapie-App zu einer signifikant stärkeren Verbesserung der Funktion im Vergleich zur Kontrollgruppe führt. Die Wirksamkeit der digitalen Therapie wird auf ihren multimodalen Ansatz zurückgeführt, der bewährte Elemente wie angeleitete und überwachte Übungen, Gamifizierung, Erinnerungsfunktionen sowie edukative Inhalte umfasst, um die intrinsische Motivation der Patienten zu fördern. Ein zentraler Erfolgsfaktor scheint die Fähigkeit der digitalen Therapie zu sein, die für den Behandlungserfolg essenzielle Adhärenz an das Heimtraining zu steigern und dadurch funktionelle Therapieergebnisse, insbesondere in Bezug auf Beweglichkeit und Funktionalität, zu verbessern – ohne dabei zu einer Reduktion der Patientenmotivation zu führen. Die digitale Therapie stellt somit eine vielversprechende Ergänzung zur klassischen handtherapeutischen Versorgung dar. Eine breitere Implementierung könnte dazu beitragen, Therapieergebnisse zu optimieren und Ressourcen im Gesundheitssystem effizienter zu nutzen. Zukünftig wäre eine digitale Plattform für Behandler wünschenswert, um den Rehabilitationsprozess gezielt zu steuern und frühzeitig therapeutische Anpassungen vorzunehmen. Angesichts der begrenzten Studienlage sind weitere Untersuchungen notwendig, um die langfristige Wirksamkeit und den optimalen Einsatz digitaler Therapieformen bei Handverletzungen zu evaluieren.
